# Harm Reduction as an Alcohol-Prevention Strategy

**Published:** 1996

**Authors:** Eric Single

**Affiliations:** Eric Single, Ph.D., is director of policy and research, Canadian Centre on Substance Abuse (CCSA), and professor of preventive medicine and biostatistics, University of Toronto. The CCSA Policy and Research Unit is an affiliate of the Centre for Health Promotion at the University of Toronto, Ontario, Canada

**Keywords:** harm reduction policy, prevention strategy, prevention goals, prevention of AOD associated consequences, illicit drug, alcoholic beverage, public health, legal regulation, AOD education, AOD availability, AODR (alcohol and other drug related) injury prevention, bar, moderate AOD use, heavy AOD use

## Abstract

Harm-reduction programs, first developed in an attempt to mitigate the adverse consequences of illicit drug use, can be applied to alcohol-prevention programs as well. In fact, the movement toward harm reduction in illicit drug prevention may be closely paralleled by a similar trend in the alcohol-prevention field. Harm-reduction approaches to alcohol aim to diminish the negative consequences of intoxication (e.g., by using special glassware that breaks into fine particles instead of sharp pieces, thus reducing the chance of injury during pub fights). Such measures may receive increased attention as public support declines for restrictions on alcohol availability and new evidence emerges on the potential benefits of moderate alcohol consumption. In addition, analyses suggest that harm reduction may be an efficient approach, because it often focuses on minimizing heavy-drinking occasions, which predict drinking problems more strongly than level of consumption.

The origins of harm reduction trace back to the efforts of a relatively small set of outspoken public health specialists grappling with the serious health threats posed by AIDS (Riley 1993; Strang and Stimson 1990). In response to prevailing ideas about illicit drug use—specifically, the idea that any amount of illicit drug use is harmful and prohibited (i.e., the zero-tolerance approach)—these specialists addressed the problem from a different angle by developing a variety of prevention measures specifically aimed at reducing the spread of HIV infection among intravenous drug users. These measures include exchanging used needles or syringes for new ones and distributing bleach kits for cleaning used needles. Because such prevention measures emphasize minimizing the harmful consequences of drug use, rather than eliminating drug use, they have become known as “harm-reduction” or “harm-minimization” programs. Although abstinence may be an eventual goal, harm-reduction measures can be applied even when this ideal is not achieved.

Generally, harm-reduction programs have been developed most thoroughly in the United Kingdom, The Netherlands, other parts of Europe, and, more recently, in Australia. In North America, harm-reduction programs are much less common, although they are gaining some ground. For example, more than 200 programs have been developed in rural and urban areas throughout Canada since syringe exchange programs began operating unofficially in 1987.

Evaluation studies show that harm-reduction programs have been successful. Such programs appear to have had an impact in reducing the spread of AIDS and other diseases without raising levels of drug use among the general population (for example, see Donoghoe et al. 1989; Stimson 1989; Buning 1990; Watters et al. 1990; Wodak 1990; Riley 1993). Despite the programs’ success—or perhaps because of it—the harm-reduction movement is now at a crucial cross-roads. The c once pt of harm reduction lacks a clear, uniformly accepted definition, yet such a definition is necessary as harm reduction garners increased attention. Not only are harm-reduction approaches being implemented in a greater number of geographical areas, but the concept also is expanding in terms of its acceptance on official levels (e.g., it is currently the basis of Canada’s Drug Strategy) and its scope. Harm reduction has extended beyond the goal of slowing the spread of AIDS and other communicable diseases (O’Hare 1992). Now programs aimed at ameliorating the consequences of both licit and illicit drug use are described as “harm reduction” (for example, see Plant et al. 1996), and the concept has been applied to alcohol-prevention programs as well. This article contends that the trend toward using harm-reduction approaches in illicit drug prevention strategies closely parallels several progressive alcohol-prevention programs.

## Applying Harm Reduction to Alcohol Use

Although harm reduction often is thought of in the context of illicit drug use, the same principles can be applied to alcohol use. In fact, harm-reduction measures are somewhat less controversial for alcohol than for illicit drugs, if only because the use of alcohol generally is socially acceptable and legal (except under particular circumstances, such as while driving, and for those who are younger than the minimum drinking age). Therefore, although harm-reduction and zero-tolerance approaches differ sharply over the emphasis given to deterrence of illicit drug use, the parallel contrast between harm-reduction and more traditional prevention approaches for alcohol is less distinct.

Until recently, public health advocates in the field of alcohol prevention have tended to stress alcohol-control measures (e.g., restrictions on the nature and extent of State monopolization of alcohol trade, limits on the number and location of off-premises sales outlets, licensing regulations, drinking-age restrictions, proscriptions against selling to intoxicated patrons, advertising and sponsorship limitations, criminal penalties for driving while intoxicated, and alcohol taxation). This focus on restricting alcohol availability is based on the well-established relationship among alcohol-control measures, alcohol consumption levels, and indicators of alcohol-related health and social problems (Bruun et al. 1975; Makela et al. 1981; Moore and Gerstein 1981; Edwards et al. 1994). As stated by Bruun and colleagues (1975), controls over alcohol availability are justified on the grounds of public health:

[O]ur main argument is well substantiated: *changes in the overall consumption of alcoholic beverages have a bearing on the health of the people in any society. Alcohol control measures can be used to limit consumption: thus, control of alcohol availability becomes a public health issue* (Bruun et al. 1975, pp. 12–13; italics in original).

In addition to alcohol-control measures, conventional alcohol-prevention approaches have stressed a preventive education component primarily focused on the negative effects of alcohol consumption. Generally, the message for all drinkers is unequivocal: Drinking less is better.

The message given through harm-reduction approaches is different, although complementary: Avoid problems when you drink. This admonition does not contradict the message that drinking less is better. Indeed, some harm-reduction approaches (e.g., the promotion of low-alcohol beverages) involve consuming less alcohol. Harm reduction differs from previous alcohol-prevention approaches, however, in that it focuses on decreasing the risk and severity of harmful consequences arising from alcohol consumption with-out necessarily decreasing the level of consumption itself. It is essentially a practical approach; success is not measured by the achievement of an “ideal” drinking level or situation (i.e., abstention or low-risk levels), but by whether the introduction of the prevention measure reduces the chance that adverse consequences will occur.

Harm-reduction approaches to alcohol are neutral regarding the long-term goals of intervention, which may or may not include abstention. The concept’s defining feature is its attempt to minimize the negative consequences of alcohol consumption in situations where people will be drinking. The fact that drinking will occur is accepted, implying neither approval nor disapproval, and the drinker is held responsible for his or her actions.

## Examples of Harm Reduction in Alcohol Prevention and Treatment

An excellent example of a harm-reduction approach to alcohol is the introduction of special glassware into pubs in Scotland. When broken, this glassware crystallizes into fine particles. Therefore, if a fight develops, the combatants cannot smash a glass against the bar and use the glass shards as a weapon (Plant et al. 1996). Further examples of harm-reduction measures to prevent adverse drinking consequences are discussed below:

### Measures That Directly or Indirectly Reduce the Consequences of Intoxication

Along with the use of special glassware, this category includes adjusting the physical structure or lay-out of drinking establishments (e.g., padding furniture and compartmentalizing space) to minimize harm if a fight breaks out. Another example is the “Nez Rouge” (“Red Nose”) program in Quebec, which is a community-based service providing two drivers (one for the drinker and one for his or her car) to anyone who has had too much to drink at a party or at a licensed establishment to be able to drive home safely (Single and Storm 1985). In addition, many American cities now provide free public transportation on New Year’s Eve and other festive occasions when heavy drinking is notorious for taking place. This category of harm reduction includes measures not specifically aimed at reducing drinking consequences, such as the introduction of airbags in cars, which decreases the number of alcohol-related traffic injuries and fatalities.

### Substitution of Less Intoxicating or Damaging Beverages

In many countries, low-alcohol beverages, such as light beers, low-alcohol wines, and even light spirits, have been introduced and promoted in recent years. These beverages can reduce alcohol intake without affecting the overall volume of drinking (i.e., liquid intake). Thus, they maintain industry profitability and serve a public health purpose simultaneously. This category of harm reduction also includes substituting beverage alcohol for potentially dangerous alternative alcohol sources. For example, the Alberta Liquor Control Board introduced special early hours for a store in downtown Edmonton to discourage severely alcohol-dependent people from drinking potentially lethal non-beverage alcohol (e.g., shoe polish). The measure was not intended to reduce alcohol consumption levels—in fact, it was expected to increase consumption of drinkable (i.e., potable) alcohol; the measure was directed solely toward reducing the adverse consequences that result from drinking nonbeverage alcohol.

### Server Training Programs

Server training also represents a harm-reduction measure in several respects. Most server training programs involve the development of policies by drinking establishments to promote moderation (e.g., quality upgrading, in which higher price brands are promoted to reduce total alcohol intake; pricing beverages with a lower alcohol content below higher strength beverages; and avoiding happy hours and other volume discounts or specials). They also may involve policies (e.g., designated driver programs) or environmental modifications (e.g., monitoring entrances to prevent underage or intoxicated people from entering) to reduce the likelihood that alcohol-related problems will occur. Staff are trained to recognize and gradually cease service to intoxicated patrons, offering low-alcohol or nonalcoholic alternatives instead. When these prevention efforts fail, servers also are trained to manage intoxicated patrons appropriately (e.g., ensuring that they have safe transportation to get home). Thus, server training attempts to reduce drinking-associated consequences without generally restricting drinking or adversely affecting the profitability of licensed establishments. In fact, evaluation studies (Geller et al. 1987; McKnight 1988; McKnight 1993; Gliksman et al. 1993; Homel et al. 1994; Saltz in press) typically have shown that establishments with server-intervention training tend to attract more customers and increase profitability as a result of introducing responsible serving practices. Server training programs currently are expanding beyond commercial establishments to include social hosts who serve alcoholic beverages. (For more information, see sidebar by McKnight, pp. 227–229.)

### Controlled-Drinking Programs

Some alcohol treatment programs do not require complete abstinence, but in-stead train participants to control their drinking. Such programs generally are aimed at drinkers whose alcohol use is becoming a cause for concern because of alcohol-related personal, employment, or health problems, rather than at severely alcohol-dependent drinkers. Providing a controlled-drinking program as a treatment alternative for people with alcohol problems might be considered a harm-reduction measure, although it has been argued that harm from drinking is eliminated, not merely reduced, if drinking is controlled successfully. In many ways, the often acrimonious debate concerning controlled drinking versus abstinence as a treatment goal for people with alcohol problems parallels the conflict between harm-reduction and zero-tolerance approaches in drug-use prevention. In each case, the former option makes allowances for (or at least tolerates) the continued use of alcohol or drugs, while the latter (i.e., abstinence and zero-tolerance approaches) aims to halt use altogether.

## Factors Contributing to a Harm-Reduction Trend in Alcohol-Prevention Programs

Most of the examples of harm-reduction measures presented in this article are relatively new and are part of a distinct trend toward prevention measures aimed at minimizing the negative consequences of drinking rather than decreasing drinking. Several factors contribute to this shift in alcohol-prevention approaches.

One factor is declining political support for controls over alcohol availability in numerous parts of the world, especially in light of reduced alcohol consumption in many countries and the erosion of international trade barriers. For example, although public opinion in Canada generally supports maintaining alcohol controls—such as alcohol monopolies, restraints on the number of retail outlets, and restrictions on advertising (MacNeil and Webster in press)—support for these alcohol control measures declined between the 1989 National Alcohol and Other Drugs Survey and the 1993 Canada’s Alcohol and Other Drugs Survey. This trend will likely continue as new evidence regarding the potential benefits of moderate alcohol consumption (see, for example, Klatsky et al. 1986; Moore and Pearson 1986; Stampfer et al. 1988; Klatsky et al. 1990; Klatsky et al. 1992; Poikolainen 1995) becomes more widely publicized. Although for most drinkers the risks involved in initiating or increasing alcohol consumption would outweigh any health benefits (Canadian Centre on Substance Abuse/ Addiction Research Foundation 1993), the beneficial effects of moderate drinking nevertheless have attracted widespread media attention. A recent study (Single et al. 1996) found that the number of deaths averted by the moderate use of alcohol actually is greater than the number of deaths attributable to alcohol in Canada; this finding will likely be the focus of publicity, even though it expresses only part of the research results.

In response to declining support for alcohol controls, policymakers may give greater consideration to measures that emphasize minimizing the consequences of drinking as opposed to measures that restrict access to alcohol. If this shift does take place, harm-reduction approaches may receive increased attention, because they often focus on preventing problems associated with heavy-drinking occasions (typically defined as having five or more drinks in a row), rather than persuading light and moderate drinkers to reduce their consumption level. The harm-reduction perspective relies on environmental controls, such as server intervention and preventive education, to convince drinkers at all levels of consumption to avoid risky drinking and to minimize any harm that may result from drinking.

In addition, empirical support exists for the focus on heavy-drinking occasions. Analyses of national survey data in Australia (Stockwell et al. 1994), Canada (Single and Wortley 1993), and the United States (Midanik et al. 1994) all indicate that it may be more efficient to focus on heavy-drinking occasions rather than level of consumption. In these analyses, the level of consumption and the number of heavy-drinking occasions were related to various indexes of alcohol problems. (In the Canadian analysis, for example, the alcohol problems examined included health complications, family discord, employment difficulties, financial strains, and drinking-related conflicts with the law.) Researchers consistently found that the number of heavy-drinking occasions more strongly predicted drinking problems than consumption level.

Furthermore, an interaction effect takes place between the number of heavy-drinking occasions and the level of consumption, with particularly high rates of alcohol problems among drinkers who generally consume low levels of alcohol but occasionally drink five or more drinks in a row. Table 1 presents data from the 1993 General Social Survey in Canada on the joint impact that the number of heavy-drinking occasions and level of consumption^1^ have on one’s likelihood of experiencing a drinking problem (Single et al. 1995). Light drinkers who drink immoderately (i.e., five or more drinks on a single occasion, for the purposes of this study) seven or more times per year show a greater likelihood of experiencing drinking problems than heavy drinkers who rarely or never drink immoderately (17 percent versus 5 or 7 percent). This finding may be associated with physical tolerance as well as the tendency for heavy drinkers to develop social supports and other mechanisms to minimize the adverse consequences of their drinking. Of course, heavy drinkers can control the harmful consequences of alcohol consumption only to a limited extent: Over time, heavy drinking greatly elevates the risk of chronic health consequences, such as cirrhosis. Nevertheless, for more acute alcohol problems (e.g., impaired driving, alcohol-related family dysfunction, or employment difficulties), relatively light drinkers who occasionally drink immoderately contribute substantially to overall problem levels.

To focus specifically on reducing the number of heavy-drinking occasions among all drinkers, programs can incorporate harm-reduction approaches, such as environmental measures (e.g., server-intervention programs and improved licensing enforcement) and preventive education (e.g., messages aimed at making intoxication and impaired driving socially unacceptable). These strategies differ from those focusing on heavy drinkers or the total volume of alcohol consumed, but both types of approaches are important. Programs specifically targeting heavy drinkers (e.g., early identification and intervention programs) undoubtedly would help reduce alcohol problems. These and other programs aimed at reducing overall levels of alcohol consumption, however, should not be adopted to the exclusion of approaches that focus specifically on heavy-drinking occasions. In fact, the findings previously described indicate that targeting preventive education to the general population may be most efficient (i.e., because people who consume alcohol at levels below those associated with alcohol dependence contribute substantially to levels of alcohol problems). Specifically, such preventive education should emphasize safe drinking limits as well as the importance of avoiding intoxication and other problem-causing behaviors, rather than a person’s over-all level of consumption.

In sum, the trend in many countries toward harm-reduction programs in illicit drugs is closely paralleled by a similar trend in alcohol prevention toward measures aimed at reducing the adverse consequences of heavy-drinking occasions, albeit for different reasons. With the erosion of political support for alcohol-control measures and the emergence of new evidence about potential health benefits of moderate drinking, this trend likely will continue. Future alcohol prevention may increasingly focus on reducing the harmful consequences of alcohol use, rather than on monitoring personal consumption levels to avoid dependence.

## Figures and Tables

**Table 1:** Percentage of Drinkers Reporting Drinking-Related Problems in the Previous Year, by Drinking Level^1^

	Percentage of Drinkers Reporting Drinking-Related Problems		
	
Number of Heavy-Drinking Occasions^2^ in Previous Year	Light Drinkers (< 1 drink per week)	Moderate Drinkers (1–7 drinks per week)	Heavy Drinkers (> 7 drinks per week)
0	2	2	7
1	8	8	5
2–6	7	10	14
7 or more	17	21	35

1 Respondents to the 1993 General Social Survey (in Canada) who consumed alcohol were asked whether their drinking had adversely affected their social life, physical health, happiness, home life or marriage, work, or finances in the previous 12 months.

2 Heavy-drinking occasions were defined as consumption of five or more drinks on one occasion.

SOURCE: Single et al. 1995.

## Footnotes

1 In this study, the cutoff points for different categories of drinking levels and heavy-drinking occasions were empirically chosen such that the highest categories represented the top decile of respondents and the lowest categories represented the lowest 50 percent of respondents, with the middle categories falling between. Therefore, “light drinkers” were defined as those who consumed less than 52 drinks per year (i.e., an average of less than 1 drink per week); “moderate drinkers” consumed 52 to 364 drinks per year (i.e., an average of 1 to 7 drinks per week), and “heavy drinkers” consumed more than 364 drinks annually (i.e., an average of more that 7 drinks per week). “Immoderate drinking” (i.e., a heavy-drinking occasion) was defined as consumption of five or more drinks on one occasion in the previous 12 months. “Alcohol problems” referred to adverse effects on social life, physical health, happiness, home life or marriage, work, or finances.
